# Ibrutinib-associated cutaneous mucormycosis due to an *Apophysomyces* species: report of a case and review of the literature

**DOI:** 10.1177/20499361241241199

**Published:** 2024-03-26

**Authors:** Trung Minh Nguyen, Eva Amenta, Lynne Chapman, Sarvari Yellapragada, Bhuvaneswari Krishnan, Jonathan Lim, Richard J. Hamill

**Affiliations:** Department of Medicine, Baylor College of Medicine, 1 Baylor Plz, Houston, TX 77030-3411, USA; Department of Medicine, Baylor College of Medicine, Houston, TX, USA; Section of Infectious Diseases, Baylor College of Medicine, Houston, TX, USA; Department of Medicine, Baylor College of Medicine, Houston, TX, USA; Section of Hematology and Oncology, Baylor College of Medicine, Houston, TX, USA; Department of Medicine, Baylor College of Medicine, Houston, TX, USA; Section of Hematology and Oncology, Baylor College of Medicine, Houston, TX, USA; Michael E. DeBakey Veterans Affairs Medical Center, Houston, TX, USA; Michael E. DeBakey Veterans Affairs Medical Center, Houston, TX, USA; Department of Pathology and Immunology, Baylor College of Medicine, Houston, TX, USA; Department of Medicine, Baylor College of Medicine, Houston, TX, USA; Michael E. DeBakey Veterans Affairs Medical Center, Houston, TX, USA; Department of Medicine, Baylor College of Medicine, Houston, TX, USA; Section of Infectious Diseases, Baylor College of Medicine, Houston, TX, USA; Michael E. DeBakey Veterans Affairs Medical Center, Houston, TX, USA

**Keywords:** *Apophysomyces*, ibrutinib, invasive fungal infection, mucormycosis

## Abstract

The use of ibrutinib, a Bruton tyrosine kinase inhibitor, has been associated with invasive fungal infections (IFIs). We describe a case of *Apophysomyces* infection associated with long-term use of ibrutinib for the treatment of chronic lymphocytic leukemia as well as perform a literature review of *Mucormycosis* infections in patients on ibrutinib. Our review found that the onset of IFI can occur within months to years of starting tyrosine kinase inhibitors. These reports provide a more complete picture of the risk of IFI while patients are on ibrutinib. Our case also demonstrates the utility of molecular techniques in the diagnosis of IFI, as the diagnosis was made using 28S rDNA/internal transcribed spacer PCR.

## Introduction

Ibrutinib, a Bruton tyrosine kinase (BTK) inhibitor, is an effective therapy in chronic lymphocytic leukemia (CLL) and mantel cell lymphoma and has increasingly replaced chemotherapy for the treatment of CLL over the last 10 years.^1,2^ This advancement in treatment has also given rise to the risk of associated invasive fungal infections (IFIs).^3,4^ Although ibrutinib is generally not considered myelosuppressive, reports have described IFI in patients who have been treated with prolonged courses of ibrutinib.^1,3,4^ In a retrospective study that investigated 566 patients receiving ibrutinib for the treatment of hematologic malignancy from 2010 to 2016, the incidence of ibrutinib-associated opportunistic infections (OIs) was estimated to be 4.7% at 5 years. Among these OI, IFI comprised 73.9% (17 cases) with aspergillus being the most common etiology (12 cases). Specifically, two cases of mucormycosis, one case of cryptococcosis, one case of blastomycosis, and one case of histoplasmosis were observed.^3^ Herein we describe the case of an elderly patient with CLL on ibrutinib who presented with a skin infection from *Apophysomyce*s, a thermotolerant fungus in the order Mucorales.

## Case report

An 81-year-old man with a history of hypogammaglobulinemia and B-cell CLL RAI stage IV with 17p deletion who was receiving therapy with ibrutinib presented to the emergency center with a 3-day history of right forearm pain and redness with a lesion described as a ‘blood blister’. He had no falls or trauma and no fevers or chills.

The patient was diagnosed with B-cell CLL stage 0 approximately 22 years ago; it had progressed to stage II 7 years ago and to stage IV 3 years ago. At that time, he was started on treatment with ibrutinib 420 mg daily. The dose of ibrutinib was subsequently titrated to 250 mg daily due to cardiac toxicity and atrial fibrillation. The patient also started receiving intravenous immunoglobulin (IVIG) for hypogammaglobinemia 5 years ago. At the time of this presentation, he was receiving IVIG monthly. He lives in Houston, Texas, has no recent travel history, and enjoys doing yard work. On physical examination, there was erythema and warmth on the right forearm without involvement of his elbow or wrist. The patient was diagnosed with cellulitis and discharged on a 10-day course of trimethoprim/sulfamethoxazole.

**Figure 1.**
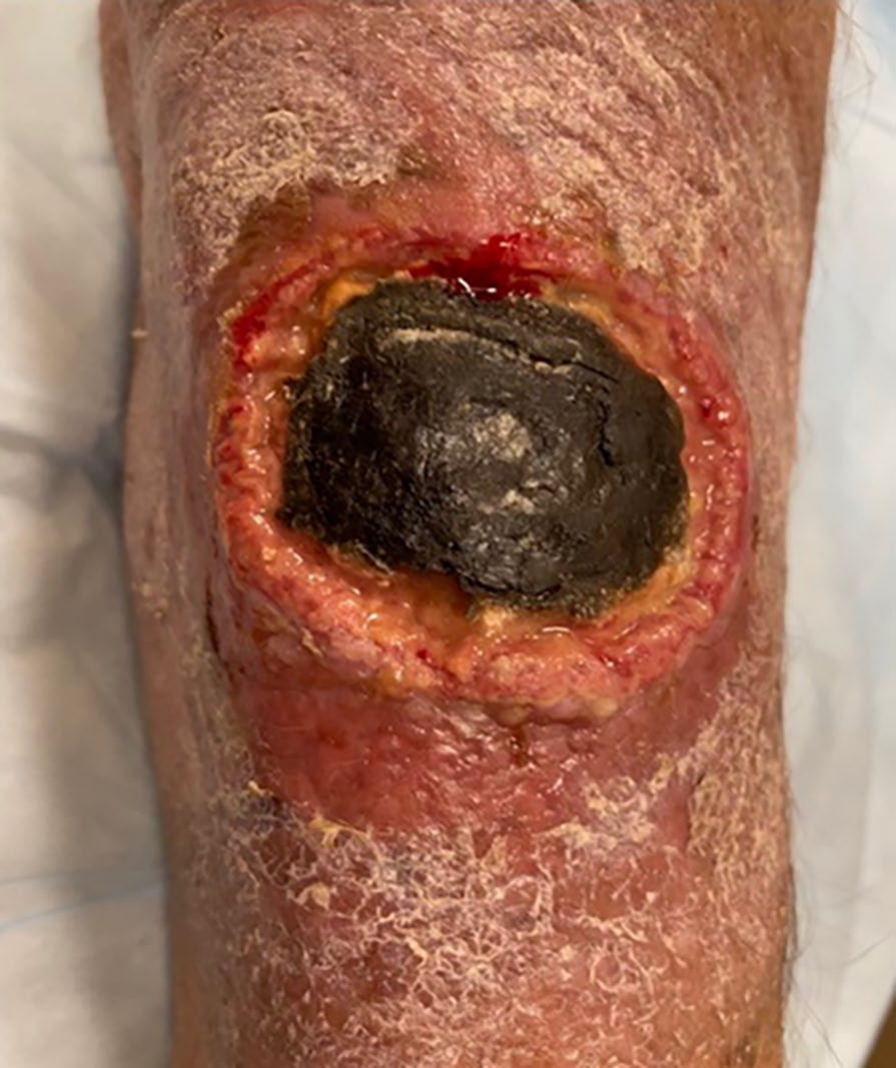
Progression of the patient’s lesion in the right forearm at the second presentation. There was a new 3.5 cm × 3.5 cm eschar with surrounding erythema that was tender to palpation.

Four days later, the patient presented again with persistent erythema and a new ulcerated lesion with eschar in the center (Figure 1). He reported subjective fevers the day prior to presentation but had no chest pain, cough, abdominal pain, shortness of breath, nausea, vomiting, diarrhea, or night sweats. He had tachycardia to 100/min with a neutrophil-predominant leukocytosis to 11,600/mm^3^ (normal range: 3500–10,000/mm^3^). Based on his presentation with mild tachycardia, worsening right forearm lesion, presumed insufficient treatment of cellulitis, and elevated white blood cell count, the patient was admitted to the hospital. He was empirically treated with cefepime 2 g Q8H and vancomycin 750 mg Q12H, while ibrutinib was held.

Throughout his hospital course, the erythema resolved but the eschar persisted. Blood culture and histological examination of biopsy specimens were negative, as were cultures for acid-fast bacteria, anaerobic bacteria, Nocardia, and fungi. Tests for *Cryptococcus*, *Histoplasma*, *Aspergillus*, HIV, methicillin-resistant *Staphylococcus aureus*, and serum 1→3,β-d-glucan were also negative. A computed tomographic scan with contrast and magnetic resonance imaging of the arm showed only cellulitis/myositis with no drainable abscess. Tissue from the lesion was sent to the University of Washington Department of Laboratory Medicine and Pathology for PCR targeting 28S ribosomal DNA (rDNA) and internal transcribed spacer (ITS) regions along with sequencing. Meanwhile, the patient was discharged with cefpodoxime 300 mg twice a day (BID) to complete a 2-week course of antibiotics for suspected concurrent cellulitis and posaconazole (300 mg BID for 1 day, followed by 300 mg daily) and infectious disease clinic follow-up in 1 week. Ibrutinib continued to be held.

At his outpatient infectious disease follow-up 1 week after discharge, the eschar had continued to grow but was non-tender. The patient and wife had been dressing the wound with meta-honey and gauze with no improvement. The patient had not yet begun to take the posaconazole since the medicine had not been obtained. He had no pain, swelling, or drainage and no fevers or chills. The patient and his wife were advised to continue local wound care with meta-honey and start the posaconazole while awaiting the molecular results.

**Figure 2.**
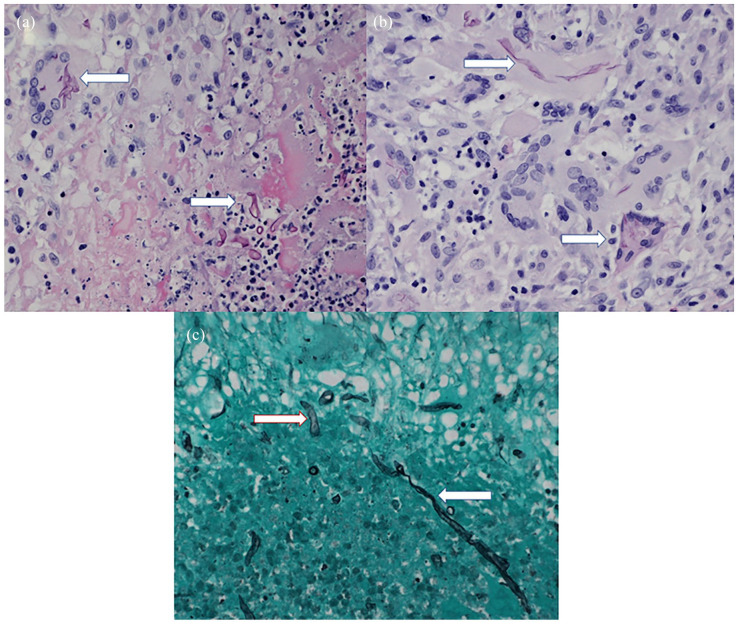
Histological sections of tissues are removed during debridement. (a) It demonstrates non-septate fungal hyphae in the central necrosis (arrow right) and within a giant cell (arrow top left) (400×). (b) It shows a higher magnification of the giant cells with the hyphal fragments (400×). (c) It shows staining of fungal hyphae with the Gomori methenamine silver stain (400×).

One day after his outpatient infectious disease follow-up, 28S rDNA and ITS PCR sequencing results returned and revealed an *Apophysomyces* species DNA. The patient was admitted and completed a 7-day course of liposomal amphotericin B (3 mg/kg daily). He was then transitioned to posaconazole 300 mg BID for 1 day, followed by 300 mg daily. The patient also underwent debridement and full-thickness skin graft. Tissue specimens from the debridement showed non-septate fungal hyphal elements consistent with a Mucorales species (Figure 2). However, specific fungal cultures had no growth. Hyphae were present in the deep advancing portions of the ulcer.

**Figure 3.**
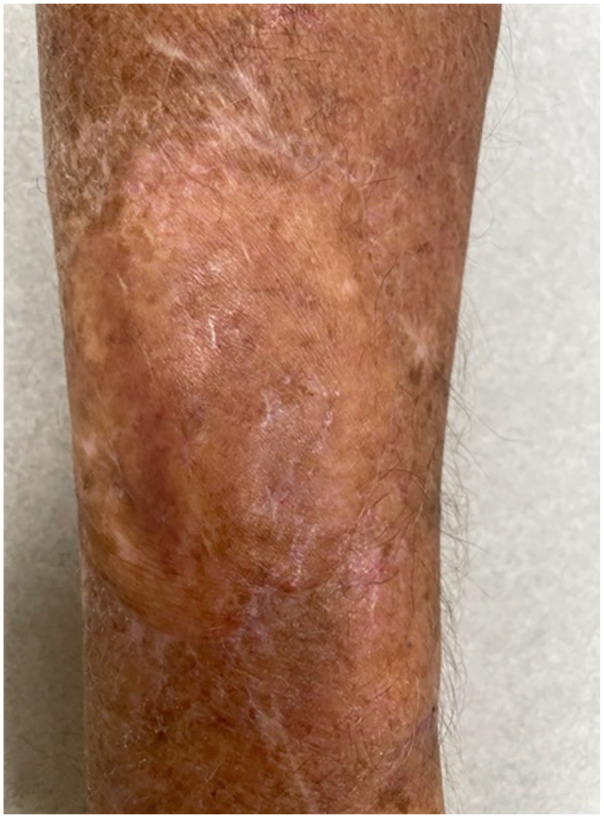
Appearance of right forearm lesion 9 months after debridement with full-thickness skin graft and 3 months of posaconazole therapy.

Posaconazole was stopped 3 months later following the resolution of the lesion. At the time of subsequent outpatient follow-up 9 months later, his arm lesion had completely healed (Figure 3).

## Discussion

### *Apophysomyces* mode of transmission

Fungi in the order Mucorales usually reside in decaying vegetation and soil. These organisms can spread during natural disaster events when people sustain lacerations or traumatic inoculation of uprooted vegetation and other debris. Several cases of *Apophysomyces* infections, specifically, have been reported following injuries suffered in tsunamis and tornadoes. During the 2004 Southeast Asian tsunami in Thailand and Sri Lanka, there were two case reports of survivors sustaining multiple soft-tissue injuries and laceration wounds who presented with large areas of full-thickness skin and fat necrosis in the buttock and thigh, respectively. Although these two cases occurred in different geographical locations (one patient was in Sri Lanka and the other was in Thailand), the patients had strikingly similar hospital courses. Despite treatment with empiric antibiotics and surgical debridement, both remained clinically infected. For these cases, histopathology and cultures eventually revealed *Apophysomyces elegans*, and the patients were treated with a prolonged course of liposomal amphotericin B resulting in gradual resolution of infection. Their wounds were also treated with further surgical debridement and skin grafting.^5,6^

Another case series reported 13 patients who developed necrotizing cutaneous mucormycosis following the 2011 EF5 tornado in Joplin, Missouri. These patients sustained a variety of wounds including fractures, blunt trauma, and penetrating wounds. On multivariate analysis, cutaneous mucormycosis was associated with penetrating trauma and an increased number of wounds.^7^ Interestingly, *Apophysomyces trapeziformis* was found in all 13 patients using 28S rDNA sequencing.^7^ Our case, however, is an unusual occurrence of *Apophysomyces* infection in the absence of known direct penetrating trauma to facilitate its seeding. Instead, our patient’s gardening hobby might have predisposed him to *Apophysomyces* infection in the context of incidental penetrating wounds or laceration.

### Mechanism of immune system impairment in BTK dysregulation

The main and most well-known mechanism of immunodeficiency in CLL patients is hypogammaglobinemia which is present in 85% of CLL patients, although defects in cell-mediated processes also occur.^8^ As a result, the predominant pathogens to which CLL patients are at risk are typically encapsulated bacteria which are primarily targeted and cleared by antibody-mediated processes.^9^ Patients with CLL may be at risk for other opportunistic pathogens, but these infections are not usually present unless the patients have received other immunosuppressive therapies such as corticosteroids, rituximab, or fludarabine. Fungal infections are rare and usually associated with recurrent neutropenia following monoclonal antibody therapies, or receipt of the agents mentioned previously.^8,10^ Our patient did not have recurrent neutropenia nor prior monoclonal antibody therapy. In the absence of other risk factors, this patient’s ibrutinib therapy likely predisposed him to an IFI.

BTKs are known to play a role in sensing infection *via* their interaction with Toll-like receptors on human dendritic cells and macrophages, promoting transcription of inflammatory cytokines and interferons.^11^ Microbes reported to be recognized by BTK include *Listeria monocytogenes*, *Staphylococcus aureus*, dengue virus, and *Aspergillus*.^10^ A substantial portion of current literature on BTK function has focused on cases of X-linked agammaglobulinemia (XLA), as various genes coding for the BTK protein are mutated in XLA, although IFI rarely occurs in patients with XLA.^11,12^ It is hypothesized that the predisposition to IFI in patients receiving ibrutinib results from a combination of defects in infection recognition, innate immune response dysregulation including off-target effects on BTK-related immune kinases, increased susceptibility to monocyte apoptosis, and impaired development and effector functions of myeloid phagocytes.^4,12^

### Literature review on the association between BTK inhibitors and mucormycosis

An increasing number of cases of BTK inhibitor (BTKi)-associated mucormycosis have been reported. Five BTKi, namely ibrutinib, acalabrutinib, zanubrutinib, tirabrutinib, and orelabrutinib, are approved for the treatment of multiple B-cell malignancies in humans.^13^ In January 2023, pirtobrutinib was also approved in the United States for the treatment of mantel cell lymphoma.^14^ By using the names of these six BTKi and by typing the keywords ‘name of BTKi’ and ‘mucormycosis’ at the same time into the search engine of the National Center for Biotechnology Information or NCBI databases, 14 case reports of BTKi-associated mucormycosis were found. In addition, another case by Castro *et al*., although not found in the NCBI databases, was also included since it was mentioned in the case by Sittig *et al.* Most noticeably, all 15 cases involved patients treated with ibrutinib as the BTKi of choice (Table 1). No cases of mucormycosis were reported in patients treated with acalabrutinib, zanubrutinib, tirabrutinib, orelabrutinib, or pirtobrutinib. In 10 cases, speciation was performed to guide therapy. These included one case of *Mucor irregularis*, four cases of *Rhizomucor* species, two cases of *Rhizopus* species, two cases of *Lichtheimia* species, and one case of *Cunninghamella bertholletiae* (Table 1). Our case is the first to report the involvement of *Apophysomyces* in the development of cutaneous mucormycosis after ibrutinib treatment.

**Table 1.** Reported cases of BTKi-associated mucormycosis.

Author, year	Age, sex	Underlying hematologic disease	Prior therapies	BTKi, dosage	Concurrent therapies	Organ affected	Time to onset since SMKi initiation	Speciation	Treatment	Outcome
Zhang *et al.*, 2022^15^	64, M	CLL	None	Ibrutinib, unknown dosage	None	Skin	Unknown	*Mucor irregularis*	Broad-spectrum antimicrobial therapy including amphotericin B cholesterol sulfate (150 mg daily), linezolid, piperacillin–tazobactam, and ganciclovir. Surgical debridement.	Improvement of the local lesion. Transferred to the local hospital to continue treatment due to financial concerns. Complete resolution at routine telephone follow-ups.
Zhang *et al.*, 2022^16^	52, M	CLL	Rituximab, fludarabine, cyclophosphamide	Ibrutinib, 420 mg daily (standard dose)	Rituximab (discontinued after four cycles)	CNS	10.5 months	*Rhizomucor pusillus*	Amphotericin B cholesterol sulfate (3 mg/kg daily followed by descending step therapy) and posaconazole, followed by posaconazole monotherapy (unspecified dosage and duration). Intracranial abscess drainage.	Improvement in local lesion at 1-month and 5-month follow-up imaging.
Hoesly *et al.*, 2021^17^	74, M	CLL	None	Ibrutinib, unknown dosage	None	Skin	Unknown	*Rhizopus* species	Surgical debridement. Posaconazole (300 mg daily) for 1 month.	Complete resolution at 1-month follow-up. No recurrence at 3-month follow-up.
Schneider *et al.*, 2021^18^	36, M	Post-kidney and pancreas transplant diffuse large B-cell lymphoma	Multiple lines of chemotherapy	Ibrutinib, unknown dosage	R-ICE protocol, rapamycin, tacrolimus, prednisolone	Liver, kidney, spleen	1 month	*Rhizomucor* species	Debridement with splenectomy. Isavuconazole and a 2-week course of liposomal amphotericin B (unspecified dosages), followed by long-term isavuconazole and ciprofloxacin (unspecified dosages and duration).	Discharged but readmitted for a persistent splenectomy lodge, treated with percutaneous drainage and necrosectomy, followed by outpatient IV antibiotic and oral isavuconazole. No recurrence of disease at 1-year follow-up.
Sittig et al., 2021^19^	72, M	CLL	None	Ibrutinib, 140 mg nightly (lower than standard dose)	Prednisone	Skin	Unknown	None	10-day liposomal amphotericin B (5 mg/kg daily), followed by posaconazole (300 mg daily) for 90 days. IV cefepime for positive *Pseudomonas in* wound culture. No surgical debridement.	Complete resolution of local lesion at 6-month follow-up.
Fehr *et al.*, 2020^20^	71, M	CLL	None	Ibrutinib, unknown dosage	Obinutuzumab, venetoclax, dexamethasone	CNS, pancreas, kidney, lung	25 days	*R. pusillus*	Broad-spectrum antibiotics. No antifungals were administered (fungal infection only discovered at autopsy).	Neurological deterioration hours after admission. Death after 4 days due to uncontrolled CNS pressure.
Grossi *et al.*, 2019^21^	70s, M	CLL	Fludarabine, cyclophosphamide, and rituximab	Ibrutinib, 420 mg daily (standard dose)	None	Liver, spleen, lungs, cervical vertebrae	1 month	*Lichtheimia corymbifera*	Liposomal amphotericin B (3 mg/kg daily) and antibiotics with meropenem and amikacin.	Death after 8 days of admission due to multiple organ failure.
Mascarella *et al.*, 2019^22^	79, M	CLL	None	Ibrutinib, unknown dosage	None	Thyroid gland	2 months	*Cunninghamella bertholletiae*	Surgical debridement. Liposomal amphotericin B (5 mg/kg daily) for 2 weeks, followed by posaconazole (unspecified dosage) for 12 weeks.	Complete resolution 8 months after discontinuation of antifungal therapy.
Pouvaret *et al.*, 2019^23^	52, F	CLL	Rituximab, fludarabine, and cyclophosphamide	Ibrutinib, unknown dosage	None	Mucormycosis of the kidney and spleen; aspergillosis of the CNS	6.5 months	*Lichtheimia* species (kidney, spleen); *Aspergillus fumigatus* (CNS)	Liposomal amphotericin (5 mg/kg daily) and isavuconazole (200 mg TID for 2 days then 200 mg daily), followed by long-term isavuconazole (unspecified dosage and duration).	Improvement of local lesions on follow-up imaging 6 weeks later. No symptoms at 9-month follow-up.
Mir *et al.*, 2018^24^	61, M	CLL	Bendamustine and rituximab	Ibrutinib, unknown dosage	Unknown	Lungs	Unknown	*Rhizopus* species	Video-assisted thoracoscopic surgery with pneumonectomy. Liposomal amphotericin B (5 mg/kg daily) for an extended course (46 days) due to surgical complications, followed by posaconazole (unspecified dosage and duration).	Wound complications and infections for months. Discharged but readmitted for viral pneumonia. Death 7 months later due to complications during the second admission.
Kreiniz *et al.*, 2018^25^	67, M	CLL	Rituximab and fludarabine	Ibrutinib, 420 mg daily (standard dose)	None	Lungs	7 months	None	Unspecified treatment targeting mucormycosis.	Developed pulmonary hemorrhage and rapid clinical deterioration. Death during the same admission.
Stein *et al.*, 2018^26^	68, M	CLL	Rituximab, cyclophosphamide, vincristine, and prednisone, fludarabine and rituximab	Ibrutinib, 420 mg daily (standard dose)	None	Skin	3 years	None	Liposomal amphotericin B (5 mg/kg daily) for 4 weeks and antibiotics with daptomycin and meropenem, followed by posaconazole (400 mg BID, unspecified duration).	Improvement of the local lesion. Discharged to a skilled nursing facility after 2 months of hospitalization.
Farid *et al.*, 2017^27^	61, M	CLL	Multiple treatments, including allogeneic peripheral blood stem cell transplant	Ibrutinib, unknown dosage	None	CNS	Unknown	*R. pusillus*	Liposomal amphotericin B (5 mg/kg daily) for 4 weeks, followed by posaconazole (unspecified dosage and duration).	Complete resolution. No sign of disease recurrence at 1-year follow-up.
Serota *et al.*, 2017^28^	79, M	CLL	None	Ibrutinib, unknown dosage	None	Sinuses	17 months	None	Liposomal amphotericin B for 7 days (unspecified dosage) and isavuconazole (unspecified dosage), followed by long-term isavuconazole (unspecified dosage and duration).	Discharged with stable condition after 17 days. Continued to have improved symptoms at 2-month follow-up.
Castro, 2016^29^	Unknown age, M	CLL	Unknown	Ibrutinib, unknown dosage	Unknown	Skin	Unknown	None	Liposomal amphotericin B (unknown dosage and duration), transitioned to posaconazole inpatient (unknown dosage and duration) due to intolerance of liposomal amphotericin B.	The overall function continued to decline despite improvement in local lesions. The patient elected to withdraw care; death was not attributed to mucormycosis.
Our case	81, M	CLL	None	Ibrutinib, 420 mg daily (standard dose) titrated to 250 mg daily	None	Skin	3 years	*Apophysomyces*	Liposomal amphotericin B (3 mg/kg daily) for 7 days, followed by posaconazole (300 mg BID for 1 day then 300 mg daily). Surgical debridement.	Posaconazole stopped 3 months after the resolution of the local lesion. Complete resolution of the local lesion at 9-month follow-up.

BID, twice a day; BTKi, Bruton tyrosine kinase inhibitor; CLL, chronic lymphocytic leukemia; CNS, central nervous system; IV, intravenous; R-ICE, rituximab, ifosfamide, carboplatin, etoposide; TID, three times a day.

These patients presented with a wide range of organ involvement including CNS, lungs, sinuses, liver, kidney, spleen, pancreas, vertebrae, thyroid gland, and skin (Table 1). The time between initiation of BTKi therapy to the onset of mucormycosis in these cases ranges from the shortest interval of 25 days to the longest interval of 3 years (Table 1). Rogers *et al.* reported that the time from ibrutinib initiation to OI development ranges from 0.36 to 52.0 months with a median time of 4.68 months.^3^ Our patient’s cutaneous mucormycosis developed 3 years after beginning ibrutinib therapy.

In addition, although our patient’s ibrutinib dose was decreased due to cardiac adverse effects, it has been shown that ibrutinib dosage is not associated with infection risk. In a retrospective study in 2018 on 378 patients who received ibrutinib for the treatment of lymphoid cancer, 11.4% of these patients developed serious infections with 37.2% of these infections involving IFI.^30^ The infection risk analysis between patients with serious infections and those without any infection showed that both the daily and cumulative ibrutinib dose was not associated with infection risk.^30^ Furthermore, it was found that the majority of patients on ibrutinib with IFI did not have the classic risk factors predisposing to fungal infection including neutropenia, lymphopenia, and corticosteroid therapy.^30^

In addition to our patient, six of the reported cases were treatment naïve prior to ibrutinib (Table 1), strengthening the association between ibrutinib initiation and mucormycosis development. Most prominently, of these six cases that had no prior therapies, four received no concurrent therapy besides ibrutinib (Table 1). Similarly, our patient received ibrutinib monotherapy for his CLL treatment, strengthening the association between the BTKi and his cutaneous mucormycosis caused by *Apophysomyces*.

### The importance of speciation and the role of 28S rDNA/ITS PCR and sequencing to characterize ‘difficult-to-identify’ fungal species

Cultures for the Mucorales are frequently negative even when there is microscopic evidence of fungal hyphae in tissue specimens. A retrospective review of 929 cases of mucormycosis from 1940 to 2003 reported a culture positivity rate of only 50%.^31^ Although histological examination can suggest the presence of these organisms, microscopic appearance is not always sufficiently accurate to differentiate among various filamentous fungi and attempts at improved culture methods for the Mucorales have not been implemented successfully in clinical microbiology laboratories.^32^ The initial histological evaluation of our patient’s biopsy was negative. The limitations posed by fungal cultures and the potential errors in identification using histologic morphologies highlight the need for alternative diagnostic methods to differentiate invasive fungal pathogens. Such differentiation has therapeutic relevance as only posaconazole and isavuconazole, among the azoles, have reliable activity against Mucorales species and are the drugs of choice for oral treatment.

16S rRNA sequencing is well established as a genetic method to identify poorly described, rarely isolated, novel, or culture-negative bacteria. This mode of sequencing is particularly valuable in bacterial phylogeny and taxonomy studies due to the presence of 16S rRNA in almost all bacteria, its unchanged function over evolutionary time, and its sequence size being large enough for analytic tests.^33^ Similar techniques incorporating 28S rRNA and ITS sequencing have been used for fungi considered to be ‘difficult to identify’.^34^ Molecular methods targeting 28S rDNA and ITS sequences to assist in the clinical identification of fungi from clinical tissue specimens are becoming more commonplace, and have been described for multiple different fungal species, including various Mucorales such as *Apophysomyces*.^35–37^

## Conclusion

This case shows that mucormycosis due to *Apophysomyces* can occur in the context of immune system impairment caused by prolonged ibrutinib treatment. Despite initial negative cultures and biopsy, this pathogen was correctly identified using 28S rDNA and ITS sequencing.

## Declarations

**Ethics approval and consent to participate:** Not applicable.

**Consent for publication:** The authors have obtained written and signed consent from the patient to publish this case report.

**Availability of data and materials:** Not applicable.
